# THE PHYSICAL AND SOCIAL ENVIRONMENTS, SOCIAL ACTIVITIES, AND SUBJECTIVE WELL-BEING: FINDINGS FROM THE K2 STUDY

**DOI:** 10.1093/geroni/igz038.1026

**Published:** 2019-11-08

**Authors:** Midori Takayama, Yoshiko Ishioka, Ikuko Sugawara

**Affiliations:** 1 Keio University, Tokyo, Japan, Japan; 2 University of Tokyo, Bunkyo, Tokyo, Japan

## Abstract

It has been pointed that the environments effect subjective well-being(SWB). However, it is still not clear what aspects of environments effect SWB among older adults and if degree on physical condition of older adults cause the difference on relations between and environments and SWB. In this study, firstly, we examined the relationship between the physical and social environments, social activities, and SWB in a sample of older Japanese. Secondly, we examined the differences on the effects of environments on SWB between older adults with lower physical functions and those with higher physical functions. We used data from locally representative longitudinal study of older adults 75±1, 80±1, and 85±1 years of age (at baseline) , which was conducted in Japan (The Keio-Kawasaki Aging Study(K2 study) ; N = 1388). Concerning the environments, we assessed the physical environments (public spaces and buildings, and accessibility) and the social environments (culture and recreation programs, and inclusive social environment). Results from covariance structure analyses showed that the accessible physical environment and the social environments were significant predictors of SWB, and showed that accessibility and the social environments influenced SWB via participation of social activities, too. Moreover, results from multiple group structural equation modeling showed that accessibility was a stronger predictor of SWB among older adults with lower physical functions, while accessibility was not a predictor among older adults with higher physical functions. The potential benefits of this approach provide a basic developing compensation model of SWB for this population of older adults.

